# miR-100-5p Promotes Epidermal Stem Cell Proliferation through Targeting MTMR3 to Activate PIP3/AKT and ERK Signaling Pathways

**DOI:** 10.1155/2022/1474273

**Published:** 2022-08-21

**Authors:** Zhe Liu, Yuan Yang, Jihui Ju, Guangliang Zhang, Ping Zhang, Pengxiang Ji, Qianheng Jin, Gaobiao Cao, Rui Zuo, Hongyu Wang, Chenghao Yu, Zhiqiang Zhang, Yingying Le, Yi Fu, Ruixing Hou

**Affiliations:** ^1^Suzhou Medical College of Soochow University, Suzhou, China; ^2^Department of Hand Surgery, Ruihua Affiliated Hospital of Soochow University, Suzhou, China; ^3^Hospital of Medical College of Yangzhou University, Suzhou Ruihua Orthopedic Hospital, Yangzhou, China; ^4^Shanghai Institute of Nutrition and Health, University of Chinese Academy of Sciences, Chinese Academy of Sciences, Shanghai, China; ^5^Department of Human Anatomy, Histology and Embryology, School of Biology and Basic Medical Sciences, Soochow University, Suzhou, China

## Abstract

Skin epidermal stem cells (EpSCs) play a critical role in wound healing and are ideal seed cells for skin tissue engineering. Exosomes from human adipose-derived stem cells (ADSC-Exos) promote human EpSC proliferation, but the underlying mechanism remains unclear. Here, we investigated the effect of miR-100-5p, one of the most abundant miRNAs in ADSC-Exos, on the proliferation of human EpSCs and explored the mechanisms involved. MTT and BrdU incorporation assays showed that miR-100-5p mimic transfection promoted EpSC proliferation in a time-dependent manner. Cell cycle analysis showed that miR-100-5p mimic transfection significantly decreased the percentage of cells in the G1 phase and increased the percentage of cells in the G2/M phase. Myotubularin-related protein 3 (MTMR3), a lipid phosphatase, was identified as a direct target of miR-100-5p. Knockdown of MTMR3 in EpSCs by RNA interference significantly enhanced cell proliferation, decreased the percentage of cells in the G1 phase and increased the percentage of cells in the S phase. Overexpression of MTMR3 reversed the proproliferative effect of miR-100-5p on EpSCs, indicating that miR-100-5p promoted EpSC proliferation by downregulating MTMR3. Mechanistic studies showed that transfection of EpSCs with miR-100-5p mimics elevated the intracellular PIP3 level, induced AKT and ERK phosphorylation, and upregulated cyclin D1, E1, and A2 expression, which could be attenuated by MTMR3 overexpression. Consistently, intradermal injection of ADSC-Exos or miR-100-5p-enriched ADSC-Exos into cultured human skin tissues significantly reduced MTMR3 expression and increased the thickness of the epidermis and the number of EpSCs in the basal layer of the epidermis. The aforementioned effect of miR-100-5p-enriched ADSC-Exos was stronger than that of ADSC-Exos and was reversed by MTMR3 overexpression. Collectively, our findings indicate that miR-100-5p promotes EpSC proliferation through MTMR3-mediated elevation of PIP3 and activation of AKT and ERK. miR-100-5p-enriched ADSC-Exos can be used to treat skin wound and expand EpSCs for generating epidermal autografts and engineered skin equivalents.

## 1. Introduction

Epidermal stem cells (EpSCs) residing in the basal layer of skin epidermis play an essential role in skin homeostasis and wound repair [1, 2]. After skin injury, the EpSCs around the wound are activated to proliferate, migrate to the wound site, and differentiate to keratinocytes to regenerate epidermis [3, 4]. Therapeutic approaches based on EpSCs and EpSC-seeded scaffold are being developed to treat extensive and chronic skin wounds, such as burn and diabetic ulcer [3–5]. In addition, EpSCs are ideal seed cells for fabricating engineered skin tissues which provide efficacious therapy in cutaneous wound repair [6, 7]. Identifying endogenous factors which can promote EpSC proliferation will be helpful for developing the wound healing agent and expanding EpSCs in vitro for clinical treatment of skin injury or skin tissue engineering.

Exosomes are small extracellular vesicles containing biological molecules, such as nucleic acids, proteins, lipids, and metabolites, which mediate intercellular communication [8, 9]. Exosomes from human adipose-derived stem cells (ADSC-Exos) have been reported to accelerate wound healing primarily by activating wound healing mechanisms through alleviating inflammatory response, promoting angiogenesis, enhancing keratinocyte and fibroblast proliferation and migration, and regulating extracellular matrix remodeling [10–13]. It has been reported that exosomal noncoding RNAs from ADSCs play important roles in wound healing. For example, exosomal lncRNA H19 from ADSCs accelerates skin wound healing through promoting fibroblast proliferation and migration [14]. ADSC-Exos promotes endothelial cell angiogenesis and wound healing through transferring miR-125a [15, 16]. Human ADSCs contain a variety of miRNAs [17]. Some of them, such as miR-21, miR-31, and miR-155, can promote wound healing through enhancing keratinocyte proliferation and/or migration [18–20]. Our previous study found that human ADSC-Exos promoted human EpSC proliferation [21]. Therefore, we propose that miRNAs in ADSC-Exos may play an important role in promoting EpSC proliferation.

In the present study, we isolated human ADSC-secreted exosomes and analyzed the miRNA profile by small RNA sequencing. miR-100-5p is one of the most abundant miRNAs in ADSC-Exos. It has been reported to promote or inhibit the proliferation of cells from different origin by targeting different molecules [22–26]. miR-100-5p promoted the proliferation of human placental microvascular endothelial cells by targeting homeodomain-interacting protein kinase 2 to activate the PI3K/AKT pathway [24]. miR-100-5p inhibited endometrial stromal cell proliferation by targeting HOXA1 to inhibit the PI3K/AKT and ERK pathways [26]. As miR-100-5p from umbilical cord mesenchymal stem cell-derived exosomes can accelerate vaginal epithelial cell proliferation [22], we proposed that miR-100-5p from ADSC-Exos might have proproliferative effect on EpSCs. We investigated the effect of miR-100-5p on human EpSC proliferation and explored the underlying mechanisms and confirmed the effect of miR-100-5p on EpSC proliferation in cultured human skin tissue using miR-100-5p-enriched ADSC-Exos.

## 2. Material and Methods

### 2.1. Isolation and Culture of Human EpSCs and ADSCs

Human skin and adipose tissues were obtained from patients (under 50 years old) who underwent the second-stage plastic surgery after treatment of skin injury through anterolateral thigh flap transplantation. EpSCs were isolated form skin tissues of 44 patients (34 males and 10 females), and ADSCs were isolated from adipose tissues of 19 patients (12 males and 7 females). The protocol was approved by the ethics committee of Ruihua Affiliated Hospital of Soochow University, Suzhou, China. EpSCs were isolated from the skin tissues as previously described [27, 28]. Briefly, the epidermis was separated from the dermis by incubating the skin tissue in 0.25% dispase II (Sigma, St Louis, USA) overnight at 4°C and digested with 0.05% trypsin for 15 min at 37°C. The cell suspension was filtered and centrifuged. The cell pellet was washed with PBS, resuspended in keratinocyte growth medium-2 (KGM2) (PromoCell, Heidelberg, Germany), and seeded in culture dishes coated with collagen IV. After incubation at 37°C for 10 min, non-adherent cells were rinsed off with PBS. The adherent EpSCs were cultured in KGM2 supplemented with 4 *μ*l/ml bovine pituitary extract, 0.125 ng/ml epidermal growth factor, 0.06 mM CaCl_2_, and 10 *μ*M Y-27632 to promote cell proliferation and inhibit cell differentiation. The second passage of EpSCs cultured in KGM2 was used in the following experiments.

ADSCs were isolated from human adipose tissues as previously described [21]. Briefly, adipose tissues were minced and incubated in 0.1% collagenase I (Sigma, Saint Louis, USA) at 37°C with constant shaking for 1 h. The cell suspension was filtered, centrifuged, and washed with PBS. The cell pellets were resuspended in DMEM/F12 medium supplemented with 10% FBS and incubated overnight at 37°C in a humidified chamber with 5% CO_2_. The adherent ADSCs were cultured in fresh complete DMEM/F12 medium. The following experiments were performed with the second to forth passage of ADSCs.

### 2.2. Characterization of ADSCs

ADSCs cultured in DMEM/F12 medium to 90%–100% confluent were further cultured in MesenCult™ Adipogenic Differentiation Medium or Osteogenic Differentiation Medium (STEMCELL Technologies, Vancouver, Canada) for 9–10 days. The cells were fixed with 4% paraformaldehyde and stained with oil red O or alizarin red S to identify adipogenic and osteogenic differentiation, respectively.

To induce chondrogenic differentiation, ADSC pellet was incubated with MesenCult™ ACF Chondrogenic Differentiation Medium (STEMCELL Technologies, Vancouver, Canada) for 21 days. The pellets were fixed in 10% formalin and embedded with paraffin and thereafter stained with Alcian blue.

### 2.3. Isolation and Characterization of Exosomes

ADSC-derived exosomes (ADSC-Exos) were isolated from cell culture medium as previously described [21]. Briefly, after the cultured ADSCs reached 80% confluence, the culture medium was replaced with serum-free medium for additional 48 h. The cell culture medium was collected and centrifuged at 300 g for 15 min. The supernatant was filtered through 0.22 *μ*m sterile membrane, concentrated using 100 KDa Amicon® Ultra-15 Centrifugal Filters (Millipore, Massachusetts, USA), and incubated with ExoQuick-TC precipitation solution at 4°C overnight. The pellet (ADSC-Exos) was resuspended in PBS. ADSC-Exo protein concentration was measured using a BCA protein assay kit (Beyotime, Shanghai, China).

The morphology of ADSC-Exos was observed under a transmission electron microscopy. The size distribution of ADSC-Exos was analyzed using ZetaView (Particle Metrix, Germany). Western blotting was performed to determine exosome-specific surface markers CD63 and Alix using primary antibodies from Abcam (Cambridge, UK).

### 2.4. ADSC-Exo miRNA Sequencing

The exosomes were isolated from cultured ADSCs from a patient who underwent the second-stage plastic surgery aforementioned. Total RNA was extracted from ADSC-Exos using the miRNeasy Serum/Plasma Kit (QIAGEN, California, USA) and qualified and quantified using a Nano Drop and Agilent 2100 bioanalyzer (Thermo Fisher, Massachusetts, USA). The small RNA library was constructed and sequenced by BGI Genomics Co. Ltd. using the BGISEQ-500 platform (BGI, Shenzhen, China).

### 2.5. Flow Cytometry

The expression of biomarkers for EpSCs and ADSCs was examined by flow cytometry. Human EpSCs or ADSCs were suspended in FACS buffer (PBS containing 1% goat serum and 5% FBS) and kept at room temperature for 1 h. EpSCs were incubated with FITC-conjugated rat anti-human *α*6 integrin antibody and PE-conjugated mouse anti-human CD71 antibody (BD Biosciences, San Jose, USA), and ADSCs were incubated with PE-labeled mouse anti-human CD34 or CD45 antibody, APC-labeled mouse anti-human CD73 antibody, or FITC-labeled mouse anti-human CD90 antibody (BD Biosciences, San Jose, USA) at room temperature for half an hour in the dark. Meanwhile, isotype-matched FITC-labeled rat IgG and PE-, FITC-, and APC-labeled mouse IgGs were served as isotype controls. The cells were washed and resuspended in FACS buffer. FACS analyses were performed on a flow cytometry (Beckman Coulter, California, USA).

Cell cycle analyses were carried out by flow cytometry. Briefly, EpSCs were transfected with miR-100-5p mimics or control miRNA (RiboBio, Guangzhou, China), MTMR3 siRNA, or control siRNA (GENEWIZ, Suzhou, China) using Lipofectamine 3000 reagents (Invitrogen, Massachusetts, USA). After 24 h, the cells were collected and fixed in 75% ethanol at 4°C overnight. The cells were then centrifuged to remove ethanol and incubated with PI/RNase Staining Buffer (BD Biosciences, San Jose, USA) for 30 min in the dark. The DNA content was analyzed using a flow cytometer (Beckman Coulter, California, USA). The sequences of miR-100-5p mimics, control miRNA, MTMR3 siRNA, and control siRNA are listed in Supplementary Table 1.

### 2.6. Cell Proliferation Assays

Human EpSCs seeded on 6 cm dishes were transfected with miR-100-5p mimics or control miRNA, MTMR3 siRNA or control siRNA, and MTMR3 expression plasmids or control vector. After 24 h, the cells were harvested and transferred into 96-well plates. Cell proliferation at the indicated time points was determined by MTT assay or BrdU incorporation assays as previously described [27]. The OD values were measured using the Multiskan™ Spectrum Microplate Reader (Thermo Fisher, Massachusetts, USA) at 490 and 450 nm, respectively.

### 2.7. Bioinformatics Analysis

The targets of miR-100-5p were predicted using miRanda, TargetScan, RNA22, and miRWalk. We further used DAVID software to perform gene ontology (GO) analysis on the overlapped target genes, and specific biological process categories were enriched.

### 2.8. Luciferase Reporter Assay

A MTMR3 3′UTR fragment containing the wild-type or mutant miR-100-5p binding site was synthesized and inserted into a pGL3-control vector (Promega, Wisconsin, USA) to construct luciferase reporter plasmid. Human HEK-293T cells were transfected with luciferase reporter plasmid together with miR-100-5p mimics or control miRNA using Lipofectamine 3000 (Invitrogen, Massachusetts, USA). A reporter plasmid encoding Renilla luciferase was cotransfected for normalization purposes. Cells were harvested 24 h after transfection, and the luciferase signals of firefly and Renilla were measured using the Dual-Luciferase® Reporter Assay System (Promega, Wisconsin, USA).

### 2.9. Immunofluorescence Staining

The expression of biomarkers of EpSCs was detected by immunofluorescence staining. Briefly, EpSCs were fixed in 4% paraformaldehyde and permeabilized with 0.5% Triton-X. After incubation with 10% goat serum for 30 min, the cells were washed and incubated with primary antibody against CK19 or *β*1 integrin (Abcam, Cambridge, UK) overnight at 4°C and then washed with PBS and incubated with fluorescence-conjugated secondary antibody for 1 h. The nuclei were stained with DAPI, and the fluorescence signals were detected under a fluorescence microscope (Olympus, Tokyo, Japan).

### 2.10. RT-PCR and Quantitative Real-Time PCR

Target gene expression was measured through RT-PCR or RT-quantitative real-time PCR (qPCR). Target miRNA expression was examined by qPCR. Briefly, total RNA was extracted from exosomes, cells, or tissues using TRIzol reagent (Invitrogen, Massachusetts, USA). Reverse transcription of mRNA and miRNA were performed using the PrimeScript™ RT Reagent Kit with the gDNA Eraser and Mir-X miRNA First-Strand Synthesis Kit (TaKaRa, Dalian, China), respectively. PCR products were separated by agarose gel electrophoresis. GAPDH was applied as endogenous control. The expression levels of target genes were semiquantified using ImageJ software (NIH Image, Bethesda, Maryland, USA). qPCR was performed using SYBR® Premix Ex Taq TM II Kit (TaKaRa, Dalian, China) on a StepOnePlus™ Real-Time PCR System (Applied Biosystems, Massachusetts, USA). Transcriptional levels for target mRNA and miRNA were normalized to GAPDH and U6, respectively. The relative expression levels were calculated using the 2^−ΔΔ*CT*^ method. The primer sequences used for RT-PCR and RT-qPCR are listed in Supplementary Table 2.

### 2.11. Measurement of Phosphatidylinositol-3,4,5-Trisphophate

Human EpSCs were lysed via repeated cycles of freeze-thaw. After centrifugation, the supernatant was collected and the phosphatidylinositol-3,4,5-trisphophate (PIP3) level was measured using the Human PIP3 ELISA Kit (J&L Biological, Shanghai, China) according to the manufacturer's instruction. The absorbance was measured at 450 nm with the Multiskan™ Spectrum Microplate Reader (Thermo Fisher, Massachusetts, USA).

### 2.12. Western Blot

EpSCs or skin tissues were lysed in RIPA lysis buffer (Beyotime, Shanghai, China) containing 0.5% protease inhibitor cocktail (Millipore, Massachusetts, USA) and 4% phosphatase inhibitors (Beyotime, Shanghai, China). The concentration of protein was measured using a BCA protein assay kit (Beyotime, Shanghai, China). Western blotting was carried out following standard protocols. The primary antibodies against MTMR3, AKT, phospho-AKT, phospho-ERK1/2 (Cell Signaling Technology, Massachusetts, USA), ERK1/2, cyclin D1/E1/A2 (Abcam, Cambridge, UK), and GAPDH (Beyotime, Shanghai, China) were used. The target proteins were detected with the SuperSignal West Pico Chemiluminescent Substrate (Thermo Fisher, Massachusetts, USA) and quantified with ImageJ software (NIH Image, Bethesda, Maryland, USA).

### 2.13. Skin Explant Culture

Human skin tissues were cultured in a 24-well transwell system as previously described [27]. Briefly, the skin tissues were cut into 0.5 cm × 0.5 cm pieces. Each piece of the skin was injected intradermally into four spots with 20 *μ*g ADSC-Exos, 20 *μ*g exosomes isolated from miR-100-5p expression plasmid-transfected ADSCs (miR-100-Exos), 20 *μ*g miR-100-Exos plus 5 *μ*g MTMR3 plasmids, or same volume of PBS and cultured in Ham's F-12 medium (Gibco, California, USA) supplemented with 10% FBS and antibiotics. The epidermal side of the skin was kept at the air-liquid interface. The medium was changed every other day. After 2 and 5 days, the skin tissues were lysed in TRIzol reagent for extracting RNA to detect miR-100-5p and MTMR3 mRNA, or fixed in paraformaldehyde and embedded in paraffin for histological and immunohistochemical assays.

### 2.14. Histology and Immunohistochemistry

Paraffin-embedded skin tissues were sectioned at 4 *μ*m using a rotary microtome (Leica, Wetzlar, Germany) and stained with hematoxylin and eosin (HE). The thickness of the epidermis was measured using ImageJ software (NIH Image, Bethesda, Maryland, USA). For immunohistochemical assay, the sections were heated in an oven at 60°C for 2 h, deparaffinized in xylene, rehydrated in an ethanol gradient (100%–80%), incubated in antigen retrieval solution containing 0.4 g/l citric acid and 3.0 g/l trisodium citrate for 20 min, and rinsed with PBS. The sections were incubated with the endogenous peroxidase blocker (MXB, Fuzhou, China) for 10 min, rinsed with PBS, and blocked with 10% goat serum for 1 h. Then, the slides were incubated with the primary antibody against *α*6 integrin, *β*1 integrin, or PCNA (Abcam, Cambridge, UK) at 4°C overnight, rinsed with PBS, and incubated with the secondary antibody from the MaxVision™ HRP-Polymer anti-Mouse IHC Kit or anti-Rabbit IHC Kit (MXB, Fuzhou, China) for 1 h. After rinsing with PBS, the sections were incubated with diaminobenzidine (MXB, Fuzhou, China), counterstained with hematoxylin, and imaged under a microscope. The positive signals of immunohistochemical staining were analyzed using ImageJ software (NIH Image, Bethesda, Maryland, USA).

### 2.15. Statistical Analysis

Each experiment was repeated at least three times and the results are expressed as mean ± SD. Statistical analysis was performed with GraphPad Prism 8.0 (GraphPad Software Inc). Unpaired two-tailed Student's *t*-test was used to assess the statistical difference between two groups. Comparisons between more than two groups were performed using one-way ANOVA. *P* < 0.05 was considered significantly different.

## 3. Results

### 3.1. Characterization of ADSC-Exos and the Profile of miRNAs in ADSC-Exos

ADSCs were isolated from human adipose tissues and cultured. Flow cytometry assay showed that these cells were positive for mesenchymal stem cell markers CD73 and CD90 and negative for vascular endothelial marker CD34 and leukocyte marker CD45 (Supplementary Figure 1(a)). These cells could differentiate to osteocytes, adipocytes, and chondrocytes, as shown by alizarin red S staining, oil red O staining, and Alcian blue staining, respectively (Supplementary Figure 1(b)). These results demonstrated the successful isolation and culture of ADSCs.

**Figure 1:**
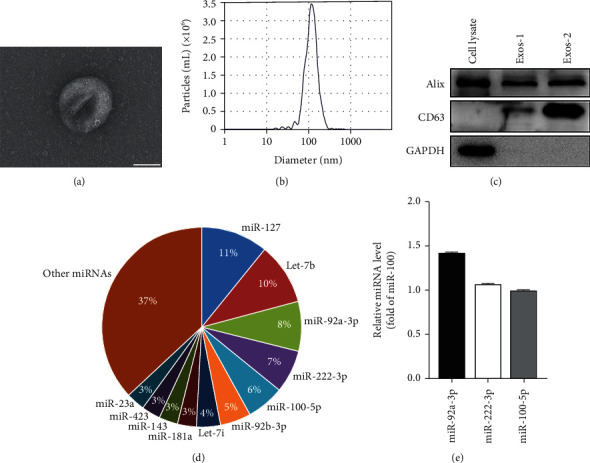
Characterization and miRNA composition of ADSC-Exos. (a) Electron micrograph of ADSC-Exos. Scale bar = 50 nm. (b) ZetaView quantification of ADSC-Exo size. (c) The expression of Alix, CD63, and GAPDH in cell lysate of ADSCs and ADSC-Exos (Exos-1 and Exos-2) was examined by Western blot. (d) miRNA composition of ADSC-Exos determined by miRNA sequencing. (e) Relative miRNA levels of miR-92a-3p, miR-222-3p, and miR-100-5p in ADSC-Exos measured by RT-qPCR.

ADSC-Exos were isolated from the serum-free culture supernatant of ADSCs. ADSC-Exos displayed a saucer-shaped morphology under transmission electron microscopy (Figure 1(a)). Nanoparticle tracking analysis showed the size of ADSC-Exos in the 100 nm range (Figure 1(b)). Western blot assay showed that ADSC-Exos expressed exosome marker proteins Alix and CD63, but not GAPDH (Figure 1(c)). These results demonstrated the successful isolation of ADSC-Exos.

By small RNA sequencing, we detected 2456 miRNAs in ADSC-Exos. The top 11 miRNAs in ADSC-Exos were presented in Figure 1(d). The expression of miR-92a-3p, miR-222-3p, and miR-100-5p in ADSC-Exos was confirmed by RT-qPCR (Figure 1(e)).

### 3.2. miR-100-5p Promotes Human EpSC Proliferation

To examine the effect of miRNA on the proliferation of EpSCs, we isolated EpSCs from human skin tissues. Flow cytometry assay showed that these cells were positive for EpSC markers *α*6 integrin, *β*1 integrin, and CK19 and negative for CD71 (Supplementary Figure 2), which was consistent with the previous studies [21, 27, 28].

**Figure 2:**
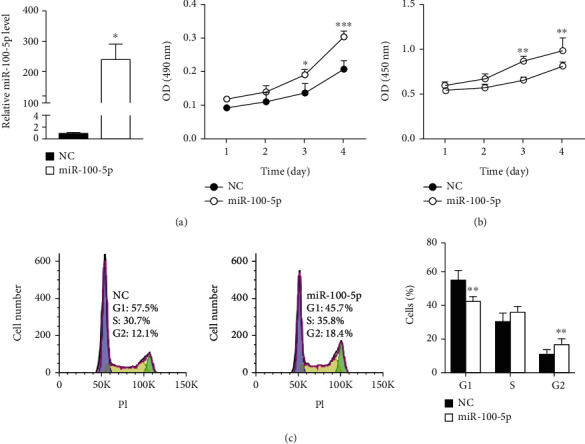
miR-100-5p promotes human epidermal stem cell proliferation. (a, b) Human epidermal stem cells (EpSCs) transfected with miR-100-5p mimics (miR-100-5p) or negative control miRNA (NC) for 24 h were examined for the miR-100-5p level by RT-qPCR or cultured for different periods of time and examined cell proliferation by MTT assay and BrdU incorporation assay, respectively. (c) EpSCs were transfected with miR-100-5p or NC for 48 h and examined for cell cycle distribution by flow cytometry. Data are shown as mean ± SD, *n* = 3. ^∗^*P* < 0.05, ^∗∗^*P* < 0.01, and ^∗∗∗^*P* < 0.001, compared with cells transfected with NC for the same period of time.

To determine the miRNA(s) from ADSC-Exos which can stimulate EpSC proliferation, we searched the literature to find out which miRNA among the top 5 miRNAs in ADSC-Exos could promote cell proliferation. Among these miRNAs, miR-127 and Let-7b have inhibitory effect on cell proliferation [29–31]; miR-92a, miR-222-3p, and miR-100-5p have been reported to stimulate or inhibit cell proliferation [22–26, 32–35]. We then examined the effect of miR-92a-3p, miR-222-3p, and miR-100-5p on the proliferation of human EpSCs. While miR-92a-3p and miR-222-3p had no effect (data not shown), miR-100-5p had stimulatory effect on EpSC proliferation. As shown in Figure 2(a), transfection of human EpSCs with miR-100-5p mimics significantly elevated the intracellular miR-100-5p level and stimulated cell proliferation in a time-dependent manner as examined by MTT assay. BrdU incorporation assay confirmed the proproliferative effect of miR-100-5p on EpSCs (Figure 2(b)). Furthermore, flow cytometry assay showed that transfection of EpSCs with miR-100-5p mimics significantly decreased the percentage of cells in the G1 phase and increased the percentage of cells in the G2 phase. The cells in the S phase had a tendency to increase (Figure 2(c)). All together, these results demonstrated that miR-100-5p promotes the proliferation of EpSCs.

### 3.3. MTMR3 Is a Target of miR-100-5p in Human EpSCs

**Figure 3:**
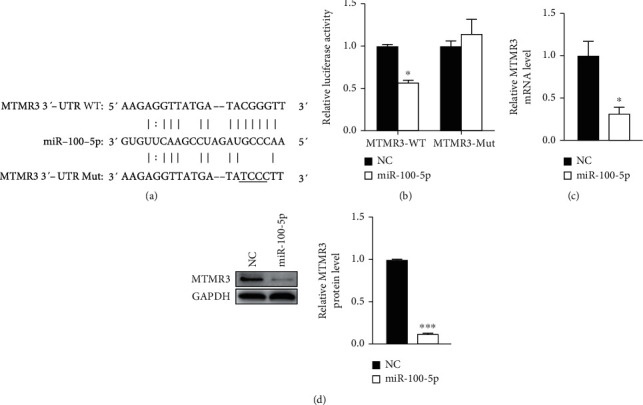
MTMR3 is a target of miR-100-5p. (a) Sequence alignment of miR-100-5p with the 3′UTR of human MTMR3 mRNA. (b) Luciferase activities in HEK293T cells cotransfected with luciferase reporter constructs containing the WT or mutated 3′UTR fragment of MTMR3 mRNA (MTMR3-WT, MTMR3-Mut) and miR-100-5p mimics (miR-100-5p) or negative control miRNA (NC) for 24 h. (c, d) Human EpSCs were transfected with NC or miR-100-5p. MTMR3 mRNA and protein levels were examined after 24 and 48 h, respectively. Data are mean ± SD, *n* = 3. ^∗^*P* < 0.05 and ^∗∗∗^*P* < 0.001, compared with cells transfected with NC.

We applied bioinformatics tools (miRanda, TargetScan, RNA22, and miRWalk) to predict the targets of miR-100-5p. Among the putative targets, 27 were overlapped. We further performed GO analysis on these 27 genes, and specific biological process categories were enriched. Among them, 7 genes (MTMR3, LAMA5, RAP1B, RRN3, IMPDH1, CDC25A, and MTOR) are associated with cell proliferation. It has been reported that MTMR3 can regulate cell proliferation in a positive or negative manner [36–41]; the other genes all positively regulated cell proliferation [42–49]. Therefore, MTMR3 may be a target of miR-100-5p in EpSC to promote cell proliferation. Sequence alignment showed that a 3′UTR fragment of the MTMR3 gene is complementary to the seed region of miR-100-5p (Figure 3(a)). To investigate whether miR-100-5p can regulate MTMR3 expression, the pGL3 promoter-based MTMR3 3′UTR reporter was cotransfected with miR-100-5p mimics or negative control miRNA to HEK293T cells. miR-100-5p mimics significantly inhibited luciferase activity of the wild-type MTMR3 3′UTR reporter but had no effect on the mutant MTMR3 3′UTR reporter (Figure 3(b)). Furthermore, transfection of human EpSCs with miR-100-5p mimics significantly reduced MTMR3 at mRNA and protein levels (Figures 3(c) and 3(d)), supporting that MTMR3 is a target of miR-100-5p in EpSCs.

### 3.4. miR-100-5p Promotes EpSC Proliferation through MTMR3

**Figure 4:**
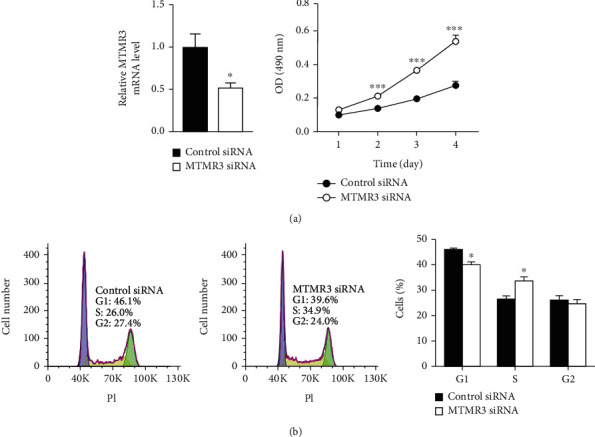
MTMR3 negatively regulates epidermal stem cell proliferation. (a) Human epidermal stem cells (EpSCs) transfected with MTMR3 siRNA or control siRNA for 24 h were examined for the MTMR3 mRNA level by RT-qPCR or cultured for different periods of time to determine cell proliferation by MTT assay. (b) EpSCs transfected with MTMR3 siRNA or control siRNA for 48 h were examined for cell cycle distribution by flow cytometry. Data are shown as mean ± SD, *n* = 3. ^∗^*P* < 0.05 and ^∗∗∗^*P* < 0.001, compared with cells transfected with control siRNA for the same period of time.

To investigate whether miR-100-5p promotes EpSC proliferation by inhibiting the expression of MTMR3, we first examined the effect of MTMR3 on EpSC proliferation by knockdown of MTMR3 in human EpSCs through RNA interference. RT-qPCR showed that the mRNA level of MTMR3 was significantly decreased after transfecting the cells with MTMR3 siRNA (Figure 4(a)). MTT assay showed that MTMR3 knockdown remarkably promoted the proliferation of EpSCs (Figure 4(a)). Meanwhile, knockdown of MTMR3 in EpSCs significantly reduced the percentage of cells in the G1 phase and increased the percentage of cells in the S phase (Figure 4(b)). These results indicate that knockdown of MTMR3 promotes EpSC proliferation by facilitating G1/S phase transition.

**Figure 5:**
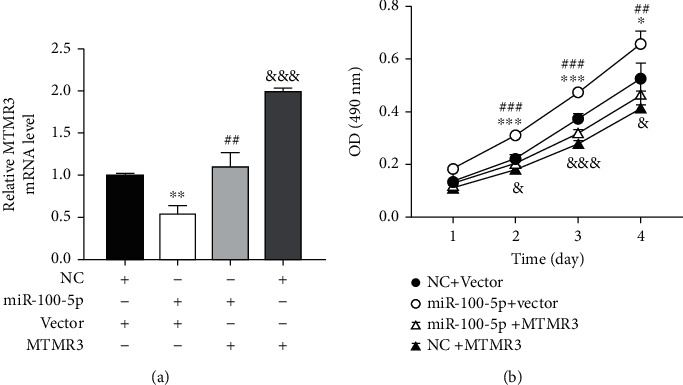
miR-100-5p promotes epidermal stem cell proliferation through downregulating MTMR3. miR-100-5p mimics (miR-100-5p) and negative control miRNA (NC) were cotransfected human epidermal cells with MTMR3 expression plasmid (MTMR3) or control vector (Vector), respectively. The MTMR3 mRNA level was examined by RT-qPCR after 24 h (a). Cell proliferation was examined at different periods of time by MTT assay (b). Data are shown as mean ± SD, *n* = 3. ^∗^*P* < 0.05, ^∗∗^*P* < 0.01, and ^∗∗∗^*P* < 0.001, comparison between cells cotransfected with miR-100-5p + Vector and cells cotransfected with NC + Vector; ^##^*P* < 0.01 and ^###^*P* < 0.001, comparison between cells cotransfected with miR-100-5p + MTMR3 and cells cotransfected with miR-100-5p + Vector; ^&^*P* < 0.05 and ^&&&^*P* < 0.001, comparison between cells cotransfected with NC + MTMR3 and cells cotransfected with NC + Vector.

Then, we checked if overexpression of MTMR3 could reverse the proproliferative effect of miR-100-5p on EpSC proliferation. As shown in Figure 5, compared with EpSCs transfected with negative control miRNA and control vector, cotransfection of miR-100-5p mimics and control vector significantly downregulated the expression of MTMR3 in EpSCs and promoted cell proliferation, cotransfection of miR-100-5p mimics and MTMR3 plasmid reversed the effect of miR-100-5p on MTMR3 expression and cell proliferation, and transfection of EpSCs with MTMR3 expression plasmid significantly elevated the MTMR3 mRNA level and inhibited cell proliferation. These results confirmed that MTMR3 negatively regulated EpSC proliferation and demonstrated that miR-100-5p promotes EpSC proliferation through downregulating MTMR3.

### 3.5. miR-100-5p Upregulates Cyclins Expression through MTMR3

**Figure 6:**
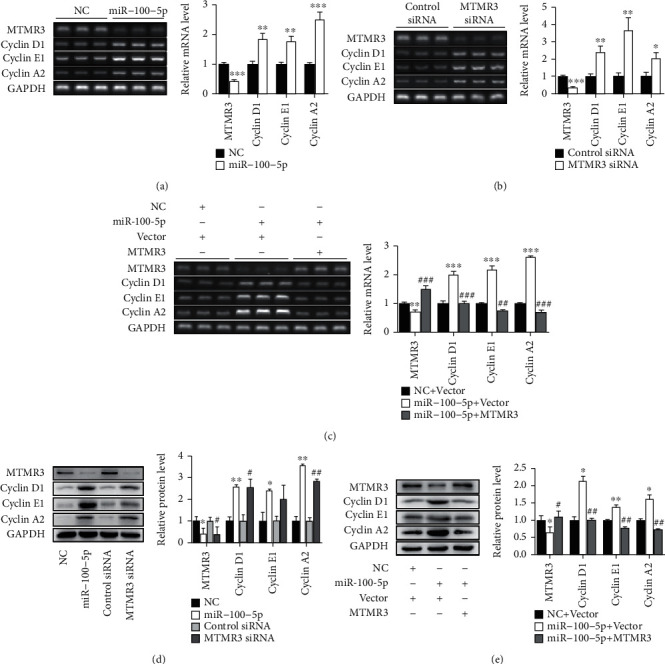
miR-100-5p upregulates cyclins expression through MTMR3. (a, b, and d) Human epidermal stem cells (EpSCs) transfected with miR-100-5p mimics (miR-100-5p) or negative control miRNA (NC); MTMR3 siRNA or control siRNA for 24 h was examined for the expression of MTMR3 and cyclins D1, E1, and A2 at mRNA and protein levels. (c, e) EpSCs cotransfected with NC and control vector (Vector), miR-100-5p and Vector, or miR-100-5p and MTMR3 expression plasmid (MTMR3) for 24 h were examined for the expression of MTMR3 and cyclins D1, E1, and A2 at mRNA (c) and protein levels (e). Data are shown as mean ± SD, *n* = 3. ^∗^*P* < 0.05, ^∗∗^*P* < 0.01, and ^∗∗∗^*P* < 0.001, compared with cells transfected with (a, d) NC, (b) control siRNA, or (c, e) NC and Vector; ^#^*P* < 0.05, ^##^*P* < 0.01, and ^###^*P* < 0.001, compared with cells transfected with (c, e) miR-100-5p and Vector or (d) control siRNA.

To explore the mechanisms involved in the regulation of cell proliferation by miR-100-5p and MTMR3, we examined the effect of these molecules on the expression of cyclins. Transfection of human EpSCs with miR-100-5p mimics significantly reduced MTMR3 and significantly elevated cyclins D1, E1, and A2 at both mRNA and protein levels (Figures 6(a) and 6(d)). The similar results were observed in EpSCs transfected with MTMR3 siRNA (Figures 6(b) and 6(d)). Furthermore, while cotransfection of EpSCs with miR-100-5p mimics and control vector downregulated MTMR3 and upregulated cyclins D1, E1, and A2 at both mRNA and protein levels, cotransfection of EpSCs with miR-100-5p mimics and MTMR3 expression plasmid reversed the expression of MTMR3 and cyclins D1, E1, and A2 (Figures 6(c) and 6(e)). These results indicate that miR-100-5p upregulates the expression of cyclins D1, E1, and A2 through inhibiting MTMR3 expression.

### 3.6. miR-100-5p Promotes EpSC Proliferation through MTMR3-Mediated Activation of PIP3-AKT and ERK Signaling Pathways

**Figure 7:**
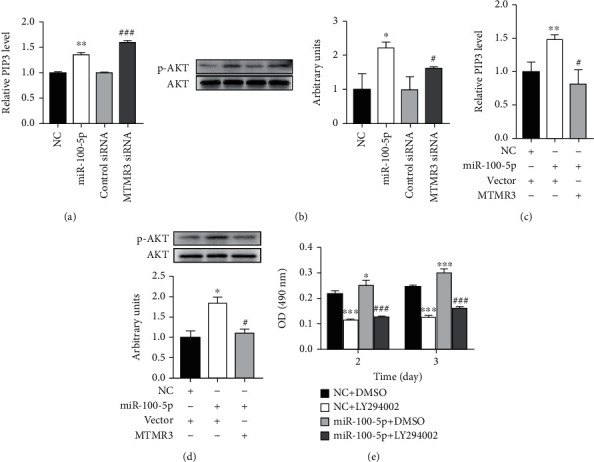
miR-100-5p promotes epidermal stem cell proliferation through MTMR3-mediated activation of the PIP3-AKT signaling pathway. (a, b) Human epidermal stem cells (EpSCs) transfected with miR-100-5p mimics (miR-100-5p) or negative control miRNA (NC); MTMR3 siRNA or control siRNA for 24 h was examined for intracellular levels of PIP3 and protein levels of p-AKT, respectively. (c, d) EpSCs cotransfected with NC and control vector (Vector), miR-100-5p and Vector, or miR-100-5p and MTMR3 expression plasmid (MTMR3) for 24 h were examined for intracellular levels of PIP3 and protein levels of p-AKT, respectively. (e) EpSCs transfected with NC or miR-100-5p for 24 h were treated with 10 *μ*M LY294002 or control solvent (DMSO) for 2 or 3 days; cell proliferation was measured by MTT assay. Data are shown as mean ± SD, *n* = 3. ^∗^*P* < 0.05, ^∗∗^*P* < 0.01, and ^∗∗∗^*P* < 0.001, compared with cells transfected with (a, b) NC, (c, d) NC + Vector, or (e) cells transfected with NC and treated with DMSO; ^#^*P* < 0.05 and ^###^*P* < 0.001, compared with cells transfected with (a, b) control siRNA, (c, d) miR-100-5p + Vector, or (e) transfected with miR-100-5p and treated with DMSO.

MTMR3 is a member of the MTMR family which are lipid phosphatases that specifically remove the 3-phosphate from phosphatidylinositol 3-phosphate (PI(3)P) and phosphatidylinositol 3, 5-bisphosphate (PI(3,5)P2) [50]. Several members of the MTMR family have been reported to negatively regulate AKT activity [51–53]. As AKT can be activated by PIP3 and plays an important role in cell proliferation [54], we investigated if miR-100-5p and MTMR3 could regulate the PIP3 level and AKT activity in EpSCs. Transfection of human EpSCs with miR-100-5p mimics significantly increased the intracellular PIP3 level (Figure 7(a)). Knockdown of MTMR3 by RNA interference also elevated the PIP3 level in EpSCs (Figure 7(a)). Cotransfection of EpSCs with miR-100-5p mimics and MTMR3 expression plasmid reversed the elevation of PIP3 by cotransfection of miR-100-5p mimics and control vector (Figure 7(c)), indicating that miR-100-5p upregulates the PIP3 level in EpSCs via MTMR3. Meanwhile, Western blot assays showed that transfection of EpSCs with miR-100-5p mimics or MTMR3 siRNA induced AKT phosphorylation (Figure 7(b)) and overexpression of MTMR3 significantly inhibited miR-100-5p mimic-induced AKT phosphorylation (Figure 7(d)). These results demonstrated that miR-100 activates the PIP3-AKT pathway through downregulating MTMR3. We further examined the contribution of the PIP3-AKT pathway to the proproliferative effect of miR-100-5p on EpSCs. Treatment of EpSCs with PI3K inhibitor LY294002, which could inhibit the conversion of PIP2 to PIP3 by PI3K, significantly inhibited cell proliferation. miR-100-5p mimics-induced EpSC proliferation was also suppressed by LY294002 (Figure 7(e)). Collectively, these results indicate that miR-100-5p promotes EpSC proliferation through MTMR3-mediated activation of the PIP3-AKT signaling pathway.

**Figure 8:**
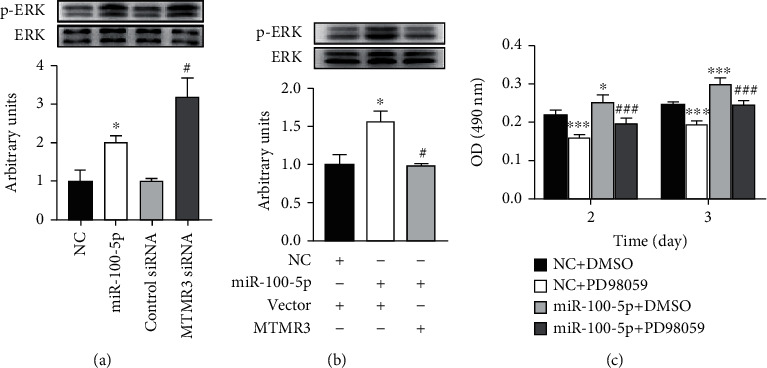
miR-100-5p promotes epidermal stem cell proliferation through MTMR3-mediated activation of ERK. (a) Human epidermal stem cells (EpSCs) transfected with miR-100-5p mimics (miR-100-5p) or negative control miRNA (NC); MTMR3 siRNA or control siRNA for 24 h was examined for ERK phosphorylation. (b) EpSCs cotransfected with NC and control vector (Vector), miR-100-5p and Vector, or miR-100-5p and MTMR3 expression plasmid (MTMR3) for 24 h were examined for ERK phosphorylation. (c) EpSCs transfected with NC or miR-100-5p for 24 h were treated with 10 *μ*M PD98059 or control solvent (DMSO) for 2 or 3 days; cell proliferation was measured by MTT assay. Data are shown as mean ± SD, *n* = 3. ^∗^*P* < 0.05, ^∗∗^*P* < 0.01, and ^∗∗∗^*P* < 0.001, compared with cells transfected with (a) NC, (b) NC + Vector, or (c) cells transfected with NC and treated with DMSO; ^#^*P* < 0.05 and ^###^*P* < 0.001, compared with cells transfected with (a) control siRNA, (b) miR-100-5p + Vector, or (c) cells transfected with miR-100-5p and treated with DMSO.

It has been reported that ADSC-Exos promoted keratinocyte proliferation through inducing ERK phosphorylation and cyclin D1/A2 expression [55]. We examined the effect of miR-100-5p and MTMR3 on the activation of ERK. Transfection of human EpSCs with either miR-100-5p mimics or MTMR3 siRNA enhanced ERK phosphorylation (Figure 8(a)), and overexpression of MTMR3 reversed ERK phosphorylation induced by miR-100-5p (Figure 8(b)), indicating that miR-100-5p activated ERK through MTMR3. Furthermore, ERK inhibitor PD98059 could abolish miR-100-5p mimics-induced EpSC proliferation (Figure 8(c)). These results demonstrated that miR-100-5p activated ERK through downregulating MTMR3, which in turn promoted the proliferation of EpSCs.

### 3.7. miR-100-5p from ADSC-Exos Promotes EpSC Proliferation in Human Skin Tissue through MTMR3

**Figure 9:**
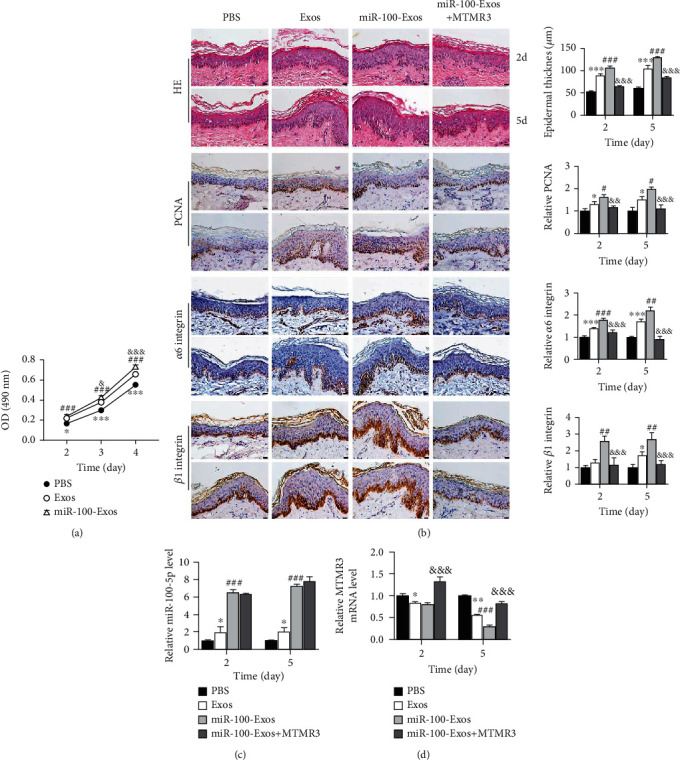
miR-100-5p promotes EpSC proliferation in the organotypic skin through MTMR3. (a) Human EpSCs were cultured with 20 *μ*g ADSC-Exos (Exos), miR-100-5p-enriched ADSC-Exos (miR-100-Exos), or same volume of PBS for different periods of time and examined for cell proliferation by MTT assay. (b–d) Human skin explants intradermally injected with PBS, Exos, miR-100-Exos, or miR-100-Exos + MTMR3 expression plasmid (MTMR3) were cultured for 2 and 5 days. Histology was examined by HE staining; cell proliferation and EpSCs were examined by immunohistochemical staining of PCNA, *β*1 integrin and *α*6 integrin. miR-100-5p and MTMR3 mRNA levels were examined by RT-qPCR. Data are presented as the mean ± SD, *n* = 3. ^∗^*P* < 0.05, ^∗∗^*P* < 0.01, and ^∗∗∗^*P* < 0.001, comparison between Exo- and PBS-treated EpSCs or skin explants; ^#^*P* < 0.05, ^##^*P* < 0.01, and ^###^*P* < 0.001, comparison between miR-100-Exo- and PBS-treated EpSCs or miR-100-Exo- and Exo-treated skin explants; ^&^*P* < 0.05, ^&&^*P* < 0.01, and ^&&&^*P* < 0.001, comparison between miR-100-Exo- and Exo-treated EpSCs or miR-100-Exos + MTMR3- and miR-100-Exos-treated skin explants. Images in (b) are representative results of 3 independent experiments. Scale bar = 20 *μ*m.

To investigate if miR-100-5p could promote EpSC proliferation in the human skin, we used miR-100-5p-enriched ADSC-Exos (miR-100-Exos) to deliver miR-100-5p. We first examined the effect of miR-100-Exos on the proliferation of EpSCs in vitro. As shown in Figure 9(a), both ADSC-Exos (Exos) and miR-100-Exos significantly stimulated EpSC proliferation; the proproliferative effect of miR-100-Exos was greater than that of Exos. We then examined the effect of miR-100-Exos on the proliferation of EpSCs in cultured skin tissues. Human skin tissue explants were intradermally injected with PBS, Exos, or miR-100-Exos and cultured for 2 and 5 days. HE staining showed that the thickness of epidermis was significantly increased at 2 and 5 days after the injection with Exos or miR-100-Exos (Figure 9(b)). Immunohistochemical staining of cell proliferation marker PCNA or EpSC markers *β*1 integrin and *α*6 integrin showed that the cells expressing PCNA, *α*6 integrin, or *β*1 integrin were located in the basal layer of the epidermis in PBS-injected skin tissue. Five days after the injection of Exos, or 2 and 5 days after the injection of miR-100-Exos, the number of PCNA-, *β*1 integrin-, and *α*6 integrin-positive cells was significantly increased in the basal layer of epidermis (Figure 9(b)). The thickness of epidermis and numbers of PCNA-, *β*1 integrin-, and *α*6 integrin-positive cells was greater and increased earlier in miR-100-Exos-treated skin tissues than in Exos-treated skin tissues. These results demonstrated that exosomal miR-100-5p promotes the proliferation of EpSCs in human skin tissue. Furthermore, intradermal injection of miR-100-Exos together with MTMR3 expression plasmid completely abolished miR-100-Exos-induced increase of epidermis thickness and numbers of PCNA-, *β*1 integrin-, and *α*6 integrin-positive cells in the basal layer (Figure 9(b)). RT-qPCR assays showed that both Exos-treated and miR-100-Exos-treated skin tissues had higher levels of miR-100-5p and lower levels of MTMR3 than PBS-treated skin tissues; miR-100-Exos-treated skin tissues had higher levels of miR-100-5p and lower levels of MTMR3 than Exos-treated skin tissues (Figures 9(c) and 9(d)). Intradermal injection of miR-100-Exos together with MTMR3 expression plasmid reversed miR-100-Exos-induced decrease of MTMR3 (Figure 9(d)). Taken together, these results indicate that miR-100-5p promotes EpSC proliferation in human skin tissues through inhibiting MTMR3 expression.

## 4. Discussion

In the present study, we found that human ADSC-derived exosomes contain high levels of miR-100-5p. miR-100-5p promoted the proliferation of human EpSCs in culture and in human skin tissue explants. Mechanistic studies revealed that miR-100-5p promoted EpSC proliferation through MTMR3-mediated elevation of PIP3 and activation of AKT and ERK.

It has been reported that ADSC-Exos promoted the proliferation and migration of keratinocytes by activating the AKT signaling pathway [56, 57]. Our previous study showed that ADSC-Exos promoted human EpSC proliferation partly through upregulating the expression of *β*-catenin, c-Myc, and cyclins D1, E1, and A2 [21] but the exosomal molecules that mediate the proproliferative effect of ADSC-Exos on EpSCs remain unclear. Human ADSC-Exos are rich in miRNAs which represent approximately 44% of all small noncoding RNA detected in ADSC-Exos [17]. By small RNA sequencing, we found that human ADSC-Exos contained multiple miRNAs. miR-100-5p was one of the most abundant miRNAs in ADSC-Exos (Figure 1). In vitro study showed that miR-100-5p promoted human EpSC proliferation in a time-dependent manner (Figures 2(a) and 2(b)). We found that transfection of EpSCs with miR-100-5p mimics decreased the percentage of cells in the G1 phase and increased the percentage of cells in the S phase and G2 phase (Figure 2(c)), which confirmed the proproliferative effect of miR-100-5p on EpSCs. Cyclins are a group of proteins that regulate genes essential for cell cycle progression by binding with various cyclin-dependent kinases (CDK). In mammalian cell division cycle, cyclin D-CDK4/6 complexes control entry into the cell cycle from quiescence and progression throughout the G1 phase. Cyclin E-CDK2 complex controls entry into the S phase. Cyclin A-CDK2/1 complexes control DNA replication and progression through the G2 phase [58]. Our study demonstrated that miR-100-5p promoted EpSC proliferation by upregulating the expression of cyclins D1, E1, and A2 (Figures 6(a) and 6(d)), which was consistent with the effect of ADSC-Exos on EpSC proliferation [21].

By bioinformatics prediction, luciferase reporter assay and miR-100-5p mimic transfection experiment, MTMR3 was identified as a direct target of miR-100-5p in human EpSCs (Figure 3). We further found that miR-100-5p promoted EpSC proliferation by inhibiting MTMR3 expression (Figure 5). MTMR3 is an inositol lipid phosphatase that specifically dephosphorylates the D-3 position of PI3P and PI(3,5)P_2_ [59]. It has been reported to be involved in multiple biological processes, including autophagy [60], cell migration [61], and promoting or inhibiting cancer cell proliferation [36–39, 41]. The molecular mechanisms underlying the regulation of cell proliferation by MTMR3 are not fully known. Yan et al. [39] reported that miR-10a induced glioma cell proliferation by inhibiting MTMR3 and enhancing *β*-catenin expression. miR-10a and MTMR3 had opposite effect on *β*-catenin expression. Wang et al. [41] reported that miR-1910-3p activated the NF-*κ*B signaling pathway by targeting MTMR3, thereby promoting the proliferation and migration of breast cancer cells. In the present study, we found that MTMR3 negatively regulated EpSC proliferation (Figures 4 and 5). Knockdown of MTMR3 in EpSCs by RNA interference significantly elevated the PIP3 level in EpSCs (Figure 7(a)). MTMR3 silencing may reduce PI(3)P dephosphorylation, which provides more substrate for PIP3 synthesis and increase intracellular PIP3 [62]. PIP3 is a key messenger in the PI3K/AKT signaling pathway to activate AKT which is involved in cell proliferation, metabolism, survival, and motility by phosphorylation of a myriad of downstream effector molecules [63, 64]. AKT activation stimulates cell proliferation through multiple downstream targets impinging on cell-cycle regulation. It promotes cyclin D expression and prevents degradation of cyclin D and cyclin E by phosphorylating glycogen synthase kinase-3 [63, 65–68]. Our study showed that knockdown of MTMR3 in EpSCs by RNA interference significantly induced AKT phosphorylation (Figure 7(b)), increased the expression of cyclins D1, E1, and A2 (Figures 6(b) and 6(d)), reduced the percentage of cells in the G1 phase, and increased the percentage of cells in the S phase (Figure 4(b)). Therefore, MTMR3 knockdown promotes EpSC proliferation through PIP3-AKT-mediated G1/S phase transition. Our study showed that overexpression of MTMR3 in EpSCs reversed miR-100-5p-induced elevation of PIP3 (Figure 7(c)), phosphorylation of AKT (Figure 7(d)), upregulation of cyclin D1, E1, and A2 expression (Figures 6(c) and 6(e)), and cell proliferation (Figure 5(b)). These results demonstrated that miR-100-5p promoted EpSC proliferation through MTMR3-mediated activation of the PIP3-AKT pathway.

It has been reported that ADSC-Exos promotes keratinocyte proliferation through inducing ERK phosphorylation and cyclin D1/A2 expression [55]. ERK activation is required for cell proliferation to proceed. It regulates G1/S transition by upregulating cyclin D1 and cyclin E transcription and indirectly regulating cyclin E-CDK2 complex formation [69, 70]. Therefore, we examined whether ERK is involved in the regulation of EpSC proliferation by miR-100-5p and MTMR3. Western blot assays showed that transfection of EpSCs with miR-100-5p mimics and inhibition of MTMR3 expression by RNA interference both significantly induced ERK phosphorylation (Figure 8(a)). Overexpression of MTMR3 in EpSCs reversed miR-100-5p-induced ERK phosphorylation (Figure 8(b)). ERK inhibitor PD98059 attenuated the proproliferative effect of miR-100-5p on EpSCs (Figure 8(c)). These results demonstrated that miR-100-5p promotes EpSC proliferation through MTMR3-mediated ERK activation.

Exosomes have recently emerged as a promising drug carrier with low immunogenicity, high biocompatibility, and high efficacy of delivery. We isolated exosomes form ADSCs with or without overexpression of miR-100-5p and compared their effect on cultured EpSCs and EpSCs in the cultured human skin. miR-100-5p-enriched ADSC-Exos had more potent proproliferative effect on EpSCs than that of ADSC-Exos. Intradermal injection of ADSC-Exos or miR-100-5p-enriched ADSC-Exos both significantly elevated the miR-100-5p level and reduced MTMR3 expression in skin tissues and increased the number of EpSCs in the basal layer of epidermis and the thickness of the epidermis. Injection of miR-100-5p-enriched ADSC-Exos together with MTMR3 expression plasmid increased MTMR3 expression and reversed the effect of miR-100-5p-enriched ADSC-Exos on EpSC proliferation (Figure 9). These data demonstrated that exosomal miR-100-5p promotes skin EpSC proliferation through MTMR3. Compared with ADSC-Exos, miR-100-5p-enriched ADSC-Exos showed stronger proproliferative effect on EPSCs in the skin. Therefore, miR-100-5p-enriched ADSC-Exos can be used to effectively expand EpSCs for basic research and skin tissue engineering. It is also a potential therapeutic reagent to promote wound healing.

In the present study, we demonstrated that both miR-100-5p and miR-100-5p-enriched ADSC-Exos could promote EpSC proliferation in vitro. The limitation is that we have not performed in vivo experiment to verify the beneficial effect of miR-100-5p on skin wound healing through stimulating EpSC proliferation. We tried to enrich miR-100-5p in ADSC-Exo by transfecting ADSCs with miR-100-5p expression plasmid, but the level of miR-100-5p in the exosomes only increased to some extent. To enhance the efficacy of ADSC-Exo-mediated delivery of miR-100-5p, it is crucial to develop a convenient and efficient method to enrich miR-100-5p in isolated exosomes.

## 5. Conclusions

**Figure 10:**
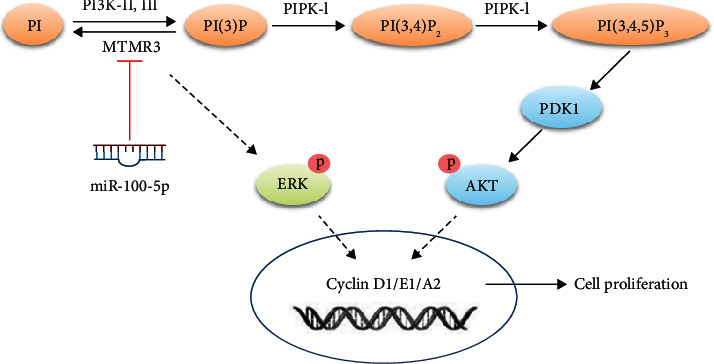
Schematic diagram of signaling pathways involved in miR-100-5p-induced proliferation of epidermal stem cells. ERK: extracellular signal-regulated protein kinase; MTMR3: myotubularin-related protein 3; PI: phosphatidylinositol; PI3K: phosphatidylinositol-3-kinase; PDK1: phosphoinositide dependent kinase-1; PIPK: phosphatidylinositol phosphate kinase.

miR-100-5p promotes EpSCs proliferation through MTMR3-mediated activation of PIP3-AKT and ERK signaling (Figure 10). miR-100-5p-enriched ADSC-Exos is a useful reagent for the expansion of EpSCs for skin tissue engineering and for skin wound treatment.

## Data Availability

The raw data used to support the findings of this study are available from the corresponding author upon request.

## Supplementary Materials

Supplementary 1 Supplementary Table 1: the sequences of miRNA and siRNA.

Supplementary 2 Supplementary Table 2: the sequences of primers for PCR and qPCR.

Supplementary 3 Supplementary Figure 1: characterization of human ADSCs.

Supplementary 4 Supplementary Figure 2: characterization of human EpSCs.
