# Supplementary material for: miR-100-5p Promotes Epidermal Stem Cell Proliferation through Targeting MTMR3 to Activate PIP3/AKT and ERK Signaling Pathways
Source: Stem Cells Int. 2022 Aug 21;2022:1474273. doi: 10.1155/2022/1474273 (PMC9421352; doi:10.1155/2022/1474273)
Supplement: Supplementary 1 — Supplementary Table 1: the sequences of miRNA and siRNA. [file 1474273.f1.docx]

**Supplementary Table 1. The sequences of miRNA and siRNA**

| **Names** | **Sequences** |
| --- | --- |
| miR-100-5p | AACCCGUAGAUCCGAACUUGUG |
| Control miRNA | UUUGUACUACACAAAAGUACUG |
| MTMR3 siRNA-F | GGAAGAUAAGGUGAAGUCA |
| MTMR3 siRNA-R | UGACAACACCUUAUCUUCCTT |
| Control siRNA-F | UUCCUUUUCCGUAUUCGCGUU |
| Control siRNA-R | CGCGAAUACGGAAAAGGAAUG |
