# Supplementary material for: miR-100-5p Promotes Epidermal Stem Cell Proliferation through Targeting MTMR3 to Activate PIP3/AKT and ERK Signaling Pathways
Source: Stem Cells Int. 2022 Aug 21;2022:1474273. doi: 10.1155/2022/1474273 (PMC9421352; doi:10.1155/2022/1474273)
Supplement: Supplementary 2 — Supplementary Table 2: the sequences of primers for PCR and qPCR. [file 1474273.f2.docx]

**Supplementary Table 2. The sequences of primers for PCR and qPCR**

| **Genes** | **Forward primer (5' → 3')** | **Reverse primer (5' → 3')** |
| --- | --- | --- |
| MTMR3 | AGTGTCAAGAGTGGCTGAAGAG | ATAGACCTCCATGCACCAAGC |
| GAPDH | AGGAACTATGACCTCGACTACG | AGTAGCTCGGTCATCATCTCCAG |
| Cyclin-A2 | TCCAAGAGGACCAGGAGAATATCA | TCCTCATGGTAGTCTGGTACTTCA |
| Cyclin-D1 | AACTACCTGGACCGCTTCCT | CCACTTGAGCTTGTTCACCA |
| Cyclin-E1 | GTCCTGGCTGAATGTATACATGC | CCCTATTTTGTTCAGACAACATGGC |
| U6 | GGCACGATACAGAGATTAGC | TGGAACGCTTCACGAATTTGCG |
| miR-100-5p | AACCCGTAGATCCGATCTTGTG |  |
| miR-92a-3p | CTCAGTAGCCAGTGTAG |  |
| miR-222-3p | CACTTGTCCCGGCCTGTAAA |  |
