# Supplementary material for: miR-100-5p Promotes Epidermal Stem Cell Proliferation through Targeting MTMR3 to Activate PIP3/AKT and ERK Signaling Pathways
Source: Stem Cells Int. 2022 Aug 21;2022:1474273. doi: 10.1155/2022/1474273 (PMC9421352; doi:10.1155/2022/1474273)
Supplement: Supplementary 3 — Supplementary Figure 1: characterization of human ADSCs. [file 1474273.f3.docx]

Supplementary Figure 1. Characterization of human ADSCs. (a) The expression of CD73, CD90, CD34 and CD45 in cultured ADSCs at the 2nd passage was examined by flow cytometry. (b) ADSCs were induced to differentiate towards osteogenic, adipogenic and chondrogenic lineages and stained with Alizarin Red (left panel), Oil Red O (middle panel) and Alcian Blue (right panel). Scale bar = 50 μm. Images are representative results of 3 independent experiments.
