# Supplementary material for: miR-100-5p Promotes Epidermal Stem Cell Proliferation through Targeting MTMR3 to Activate PIP3/AKT and ERK Signaling Pathways
Source: Stem Cells Int. 2022 Aug 21;2022:1474273. doi: 10.1155/2022/1474273 (PMC9421352; doi:10.1155/2022/1474273)
Supplement: Supplementary 4 — Supplementary Figure 2: characterization of human EpSCs. [file 1474273.f4.docx]

(c)

Supplementary Figure 2. Characterization of human EpSCs. (a) Morphology of cultured human EpSCs under a light microscope. Scale bar = 100 μm. (b, c) The expression of EpSC biomarkers α6 integrin, CD71, β1 integrin and CK19 in cultured EpSCs at the 2nd passage was examined by flow cytometry (b) and immunofluorescence staining (c), respectively. Scale bar = 50 μm. Images are representative results of 3 independent experiments.
